# Do honey bee (*Apis mellifera*) foragers recruit their nestmates to native forbs in reconstructed prairie habitats?

**DOI:** 10.1371/journal.pone.0228169

**Published:** 2020-02-12

**Authors:** Morgan K. Carr-Markell, Cora M. Demler, Margaret J. Couvillon, Roger Schürch, Marla Spivak

**Affiliations:** 1 Department of Entomology, University of Minnesota, Falcon Heights, Minnesota, United States of America; 2 Department of Molecular Biology and Genetics, Cornell University, Ithaca, New York, United States of America; 3 Department of Entomology, Virginia Tech, Blacksburg, Virginia, United States of America; Universitat Leipzig, GERMANY

## Abstract

Honey bee (*Apis mellifera*) colonies are valued for the pollination services that they provide. However, colony mortality has increased to unsustainable levels in some countries, including the United States. Landscape conversion to monocrop agriculture likely plays a role in this increased mortality by decreasing the food sources available to honey bees. Many land owners and organizations in the Upper Midwest region of the United States would like to restore/reconstruct native prairie habitats. With increasing public awareness of high bee mortality, many landowners and beekeepers have wondered whether these restored prairies could significantly improve honey bee colony nutrition. Conveniently, honey bees have a unique communication signal called a waggle dance, which indicates the locations of the flower patches that foragers perceive as highly profitable food sources. We used these communication signals to answer two main questions: First, is there any part of the season in which the foraging force of a honey bee colony will devote a large proportion of its recruitment efforts (waggle dances) to flower patches within prairies? Second, will honey bee foragers advertise specific taxa of native prairie flowers as profitable pollen sources? We decoded 1528 waggle dances in colonies located near two large, reconstructed prairies. We also collected pollen loads from a subset of waggle-dancing bees, which we then analyzed to determine the flower taxon advertised. Most dances advertised flower patches outside of reconstructed prairies, but the proportion of dances advertising nectar sources within prairies increased significantly in the late summer/fall at one site. Honey bees advertised seven native prairie taxa as profitable pollen sources, although the three most commonly advertised pollen taxa were non-native. Our results suggest that including certain native prairie flower taxa in reconstructed prairies may increase the chances that colonies will use those prairies as major food sources during the period of greatest colony growth and honey production.

## 1 Introduction

Many insect populations around the world have faced rapid declines in recent decades due to human-induced landscape changes, including massive increases in the area of land devoted to monocrop agriculture [1,2,3,4]. Declines in bee populations in particular have raised alarm because of the essential pollination services that bees provide [5,6], contributing to the global economy and improving human nutrition by making diverse fruits and vegetables cheaper to grow [5]. The most widely managed crop pollinator species, *Apis mellifera* L., the European honey bee, contributes an estimated $14 billion yearly in pollination services in the United States alone [7]. In recent years, beekeepers have seen increased honey bee colony mortality in several regions, including the United States [8,9].

While exposure to pathogens, parasites, and pesticides undoubtedly contribute to high colony mortality, poor nutrition likely plays a key role [6,9,10,11,12,13,14]. Like all bee species, honey bees require both pollen, their primary source of proteins and lipids, and nectar, their primary source of carbohydrates [15,16,17]. In temperate regions, colonies may need to collect an estimated 25 kg of pollen [17,18] and potentially over 300 kg of nectar [19] to function during the summer and survive the cold winters. In addition to quantity, diet quality is also very important. Diverse pollen sources help honey bees combat pathogens and parasites [20,21,22,23] and increase their ability to detoxify pesticides [24]. Colonies living in the temperate zone must respond to frequent changes in the species of blooming flowers from spring to fall [25,26] and may experience periods of dearth where temperatures remain high but few rewarding flowers bloom [27,28].

Many groups around the world are interested in helping maintain healthy bee populations by planting flowers for bees [29,30,31]. Simultaneously, many organizations are more broadly interested in restoring native habitats that had been converted to agriculture or other human uses [32]. Before European colonization, the Upper Midwest of the United States mainly consisted of prairie lands, defined as temperate grasslands with a moderate rainfall and deep-rooted perennial forbs [33,34]. Today less than 2% of the original prairies remain [34], and there is great interest in restoring native prairie habitats [35]. Unfortunately, governments and organizations interested in helping bees often have limited information about how different land management schemes [36] or seed mixes [29] will affect bee foraging success. Prairie restoration projects are very likely to benefit native bee species, especially bees that specialize on prairie flowers [37,38,39]. It is less clear to what extent non-native honey bees will be attracted to and use patches of flowers in reconstructed prairies.

Honey bees’ unique life cycle and foraging strategy may affect their use of flowering resources in prairies. Honey bees are generalist foragers that have a very wide foraging range, with most foraging trips occurring within 4 km of the nest [40,41,42,43] but some trips as far as 14 km away [44,45]. Honey bee colonies contain thousands of foragers that can communicate with each other about the locations of the most rewarding patches of flowers using a signal called a waggle dance, a behavior unique to bees in the genus *Apis* [26,44]. This signal involves repeated figure-eight runs in which the dancer waggles her abdomen back and forth during the straight middle portion of the figure-eight, called the waggle run. The waggle run provides dance follower bees with a vector containing both the direction of the flower patch relative to the azimuth of the sun and the distance to the patch [44]. Foragers will only dance to advertise the locations of flower patches that they perceive as profitable (having a favorable ratio of nutrients gained to energy expended) [46]. These characteristics of honey bee foraging behavior may lead colonies to focus on the densest patches of flowers and ignore sparser flowers within non-rewarding grasses, as is common in reconstructed prairie habitats. However, the density and size of flower patches in prairies change across the season as different species of flowers bloom. Even if honey bee foragers only perceive flowers in prairies as profitable resources during part of the foraging season, access to prairies may boost colony health by supplying nectar or pollen when there is a dearth of non-prairie food sources [47].

The honey bee waggle dance provides us with a window into how honey bee foragers perceive the resources that they encounter in the landscape around their hives [36]. At the level of the colony, the proportion of dances advertising flower patches in a given habitat serves as a measure of the decision-making process that allocates foragers among habitat types based on their relative profitability [36]. At the level of the individual, if a forager brings back food from a particular species of flowers and dances to advertise the site that she visited, it indicates that she perceived those flowers as sufficiently profitable to recruit nestmates. It is currently feasible to determine which flower taxon was advertised in a dance from the pollen that the dancer carried but not from nectar carried by dancers [48,49]. In addition, characteristics of dances advertising nectar sources can give more nuanced information about the perceived profitability of the resource. The total number of waggle runs that a nectar dancer performs in a dance is correlated with her assessment of the profitability of the resource advertised [46]. This relationship has been demonstrated multiple times with artificial sugar-water feeders [50,51,52], but so far has not been demonstrated with pollen or pollen substitutes ([53] but see [54]). Multiple studies have also shown that colonies tend to advertise sites at greater distances in order to find profitable nectar sources during dearth periods [27,40,42,55].

Therefore, we took advantage of the information in honey bee waggle dances to answer two main questions about how honey bee colonies perceive flowers in prairie habitats: first, is there any part of the season in which the foraging force of a honey bee colony will devote a large proportion of its recruitment efforts (waggle dances) to pollen or nectar sources within prairies? To better understand seasonal changes in the proportion of dances advertising prairies, we asked two additional questions: 1) During times of year when a large proportion of nectar dances advertise sources within prairies, do dances for prairie sites include more waggle runs and thus indicate a higher perceived profitability than dances for sites outside of prairies, and 2) Is there a seasonal change in the average distance of advertised nectar sources? For our second main question, we asked whether honey bee foragers will advertise patches of native prairie flowers as high-quality pollen sources, and, if so, which taxa will they advertise? To answer these questions, we placed honey bee colonies in glass-walled observation hives with access to two large, reconstructed prairies. The glass-walled hives allowed us to record the dances performed by members of these colonies throughout the summer and early fall. From these recordings we decoded the direction and distance information within the dances, mapped them as probability density distributions using a Bayesian modeling approach [56], and determined what percentage of the dances advertised sites within prairies during different parts of the season. At sites with a seasonal change in the proportion of dances for food sources in prairies, we also quantified waggle runs per dance to determine if the bees perceived the prairie flowers as more profitable compared to flowers outside of prairies. We also explored whether there were seasonal changes in the average distance of advertised nectar sources. Finally, to both map and identify the taxa that the foragers considered to be profitable pollen sources, we captured a subset of dancing bees who were carrying pollen loads and identified the pollen source using microscopy and DNA barcoding.

## 2 Materials and methods

### 2.1 Permits and permissions

This study required no permits because we did not sacrifice any bees and did not collect any material from flowers listed as threatened or endangered by the state of Minnesota or the United States federal government. We obtained permission from Carleton College to conduct flower surveys along specified transects through their Cowling Arboretum reconstructed prairies, and we obtained permission from Belwin Conservancy to conduct flower surveys and collect flowers within their reconstructed prairies.

### 2.2 Study sites

**Fig 1:**
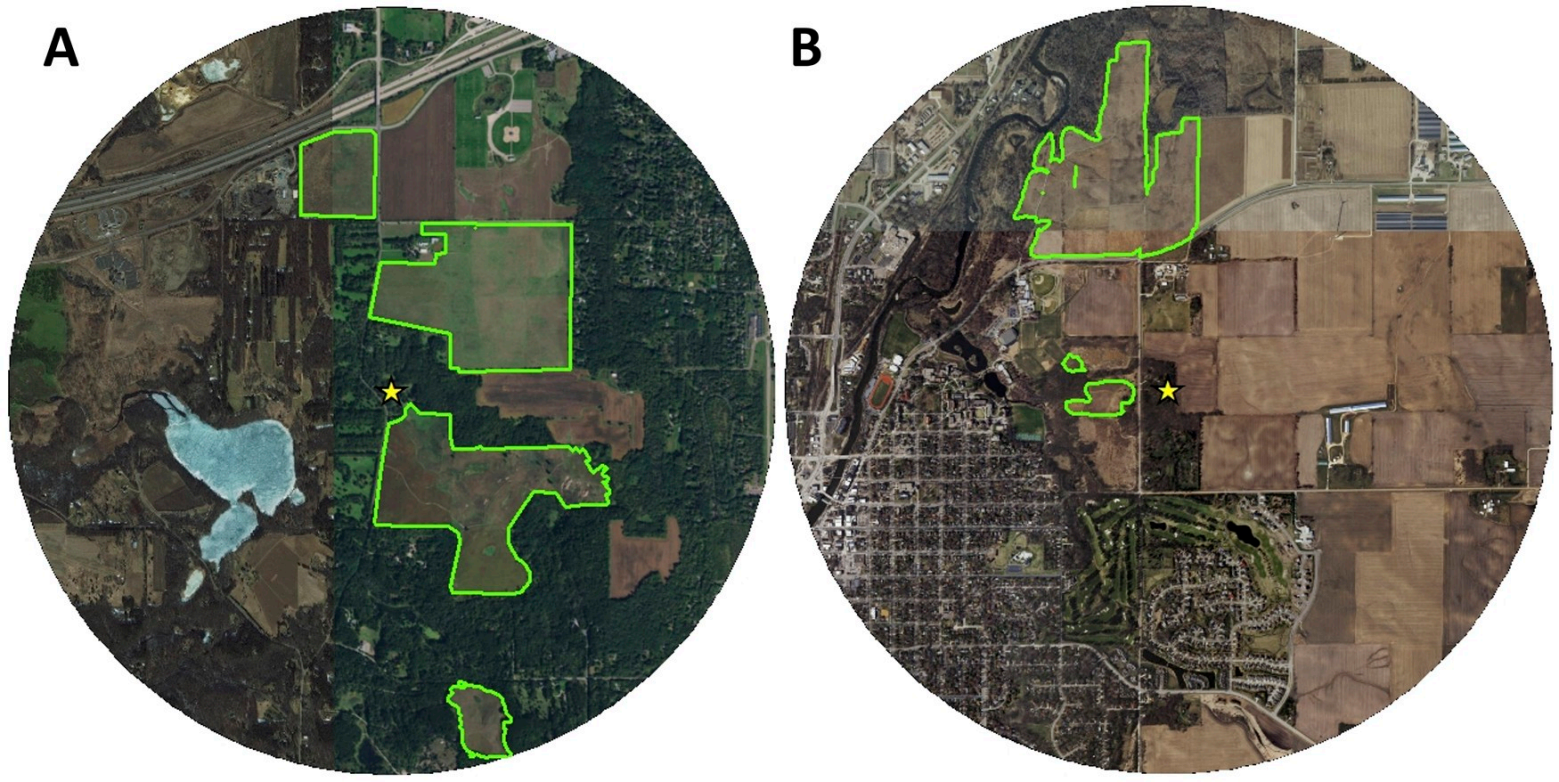
Study sites. A) Belwin Conservancy. B) Carleton College’s Cowling Arboretum. Green outlines show the edges of the restored prairies at each site. We modified prairie edges at Cowling Arboretum (Carleton College) slightly from 2010 planting unit shapefiles created by Wei-Hsin Fu and Nancy Braker using ArcGIS Version 10.0. Yellow stars represent observation hive locations. Maps extend 2 km around observation hives. World Imagery base map layer sources: Esri, DigitalGlobe, GeoEye, i-cubed, USDA FSA, USGS, AEX, Getmapping, Aerogrid, IGN, IGP, swisstopo, and the GIS User Community.

We placed colonies at two sites for this study: Belwin Conservancy (http://www.belwin.org/) in Afton, Minnesota (UTM 15N: 516102 E, 4976403 N; Latitude/Longitude: 44.940869° N, 92.795909° W) and near Carleton College’s Cowling Arboretum (https://apps.carleton.edu/campus/arb/) in Northfield, Minnesota (UTM 15N: 488949 E, 4923159 N; Latitude/Longitude: 44.461653° N, 93.138922° W; Fig 1). We observed colonies from June 9th to September 1st in 2015 and May 16th to June 24th in 2016 at Belwin Conservancy and from June 4th to September 4th in 2017 at Carleton College. Belwin Conservancy is a non-profit organization dedicated to preserving and restoring native ecosystems, including 300 acres of reconstructed tallgrass prairie, and we placed colonies between the two largest continuous patches of prairie land there (less than 2 km from the farthest edges of all Belwin prairie lands). At the Cowling Arboretum site, we placed colonies at a residence across the street from one patch of reconstructed tallgrass prairie where they had access to 160 acres of reconstructed prairie within 2 km. Both sites were planted with native prairie forbs and grasses (ranging from 5–40 years ago depending on the site and parcel of land) and are managed by a combination of periodic burning, grazing by bison (at Belwin Conservancy), and targeted removal of certain invasive species (ex. *Digitalis lanata* at Belwin Conservancy). We mapped the edges of the contiguous areas of prairie using ArcMap shape files provided by Belwin Conservancy (N. Phillips, personal communication) and Carleton College (W.-H. Fu and N. Braker, personal communication) that we modified slightly based on satellite images of the sites from ESRI (www.esri.com) using ArcGIS Desktop [57]).

### 2.3 Floral surveys

Once every two weeks during the colony observation periods, we conducted floral surveys in each of the contiguous areas of reconstructed prairie surrounding the colonies. Surveys consisted of identifying all blooming inflorescences in meter square quadrats. We placed the quadrats at ten randomly selected distances (randbetween function, Excel) along 200-meter transects near paths through each parcel of prairie land. When we noticed that certain blooming flower species were abundant in the prairies but missing from our quadrat counts we recorded those species as blooming on that sampling date. We identified blooming forbs as specifically as possible, in almost all cases to species.

### 2.4 Honey bee colony management

#### 2.4.1 Observation hives and shed

Wooden sheds protected the observation hives from the wind and direct sunlight. Florescent or LED lights illuminated the dances. We housed colonies in Ulster-style observation hives (Mann Lake, https://www.mannlakeltd.com/observation-hive) with a box on the bottom that holds 5 frames of beeswax comb and a single visible frame between two pieces of glass on the top. We modified the observation hives to reduce the space around the visible frame to approximately one bee space (~1 cm, [58]) to discourage workers from forming multiple layers and obscuring each other from view. We sealed the lower hive entrance and drilled a 2.54 cm-diameter hole through the wood halfway up the visible frame so that a plastic tube could be inserted through the wall of the observation hive shed and into the hive, allowing foragers to enter and exit only to and from the visible frame. Given that foragers prefer to dance close to the hive entrance [26,59], we added barriers so that foragers needed to travel farther into the hive to reach areas not visible to the cameras, ensuring that the dance floor would be recorded. To prevent returning foragers from descending from the visible frame to the frames below within the first 34 cm of the entrance, we used steel hardware cloth (0.32 cm spacing). To prevent foragers from moving to the opposite side of the visible frame within 34 cm of the entrance, we used metal barriers (Mann Lake, https://www.mannlakeltd.com/metal-frame-rests-10-pack).

#### 2.4.2 Colony management

We placed three colonies at each site during each year of observation. We started all colonies from 2-lb packages of workers with young, mated “Italian” queens (derived mainly from the European subspecies *Apis mellifera ligustica*) purchased from a commercial beekeeper in northern California (https://www.ohbees.com/). To make sure that all colonies had a similar sized foraging force, food stores, and brood-rearing needs, we filled each observation hive with a queen, approximately 6,000–8,000 workers, two beeswax frames filled with capped brood (pupae), one frame of uncapped brood (larvae), one frame of honey/pollen, and two drawn-out empty wax frames.

We inspected colonies once per week for signs of disease, queen problems, and/or overcrowding. When colonies appeared too crowded, we removed frames of brood. When the queen needed more space to lay eggs, we removed frames of food. When we removed these frames, we replaced them with empty, drawn comb. Because foragers appeared to prefer to dance on frames with a constant cover of other workers, whenever most of the capped pupae had emerged as adults from the upper, visible frame, we switched that frame with a brood frame from below, encouraging more workers to spend time on the visible frame. In two cases where inspections showed that the queen had died or stopped laying eggs (both at the Cowling Arboretum site in 2017), we replaced the queen with a new, mated “Italian” queen. We conducted inspections at the end of the final observation during each week to allow colonies time to adjust to any changes before the next week’s observations began. When not video recording, we kept the glass walls of the observation hives covered with foam insulation panels to help the colonies thermoregulate.

### 2.5 Video recording

We video-recorded the dance floor (area of the wax comb near the entrance where most dances occur) on one day per week at Belwin Conservancy and two days per week at Cowling Arboretum with 50-minute recordings per colony per day. The days alternated between morning recordings (8:00–12:00) and afternoon recordings (13:00–17:00), and we recorded the colonies in a random order each day. At the Belwin Conservancy site, we made all recordings with a SONY HDR-CX440 camera. At the Cowling Arboretum site, we made all recordings with a SONY FDR-AX33 camera. In both cases, we placed cameras approximately 75 cm away from the dance floor and set them to focus on an area covering approximately 30 cm wide x 25 cm high adjacent to the colony entrance. During recording, an observer pointed out all dancers and stated whether they had visible pollen loads.

### 2.6 Extracting spatial information from waggle dance communications

#### 2.6.1 Selecting waggle dances to analyze

To select approximately equal numbers of dances from all colonies and all weeks during the foraging season, we decoded the first 10 dances for nectar and the first 10 dances for pollen. We categorized dancers with pollen loads as pollen dancers and dancers without pollen loads as nectar dancers. It is possible that some of these dances could have been advertising water or resin sources. However, under normal circumstances only a small minority of foragers collect water [26,55,60] or resins [61,62,63]. Thus, it is very likely that most waggle dancers without loads on their hind legs were advertising nectar sources and most dancers with loads on their hind legs were advertising pollen sources. It is worth noting that dancers advertising pollen may have also collected nectar during the same trip, probably from the same species of plants. Most videos (mean of 57.5%) contained at least 10 dances advertising nectar sources. Less than half of videos contained 10 dances advertising pollen sources (mean of 22.5%). In videos with fewer than 10 dances advertising one of the two food resources, we decoded all dances for that resource for an average of 8.4±0.4 dances for nectar and 4.7±0.3 dances for pollen per video at Belwin Conservancy and 8.3±0.5 dances for nectar and 6.8±0.5 dances for pollen per video at Carleton College.

#### 2.6.2 Decoding waggle dances

Mapping waggle dances requires first measuring the average angle and duration of the waggle runs in each dance. We measured these two variables according to the methods of Couvillon et al. [64], except that Quicktime 7 was used to calculate the duration of each waggle run rather than FinalCutExpress. Once we observed a dance, we selected a bout of at least 6 consecutive waggle runs and avoided decoding the first and last waggle runs of this bout. This allowed us to calculate the average angle and duration of four consecutive waggle runs, which has been shown to tightly correlate with the average angle and duration of all waggle runs in a dance [64]. Because waggle dancers adjust their dance angles relative to the force of gravity, angles were measured relative to vertical plumb line references (white upholstery thread attached to tungsten weights hung from nails in the wood at the top of the visible frame and spaced 10 cm apart). The azimuth of the sun during each dance was calculated using the R subroutine SunPosition (as per Schürch et al. [56]) with an added offset of 5 hours from Greenwich Mean time (Central Standard Time during daylight savings).

#### 2.6.3 Simulating probability density clouds based on information decoded from dances

Because variation among dancing bees and among waggle runs within a dance makes the exact location of an advertised flower patch uncertain, we mapped decoded dances using estimates of this uncertainty according to the methods of [56]. For each decoded dance, we used a Bayesian linear calibration model to generate 1000 distances centered around the average distance that the dance’s duration would indicate. We determined the variance using a set of calibration data from training honey bee foragers to visit sugar-water feeders at different distances and decoding their dances. Because the calibration data from [56] did not show a significantly different relationship between waggle run duration and distance from similar data that we collected at Belwin Conservancy in September of 2015 (intercept: t(122) = 0.825, p = 0.410; slope: t(122) = 0.618, p = 0.537), we combined both sets of calibration data for generating estimated distances. Together the combined calibration dataset shows a relationship between dance duration in seconds (d) and distance in meters (D) of:

D=823.7232d–226.6063

In addition, we used the average angular dispersion of calibration data from Belwin Conservancy (von Mises distribution, kappa = 35.4 based on the spread of dances for the farthest feeder at Belwin Conservancy) to generate 1000 headings, centered around the average heading of the dance. By combining the estimated distances and headings, we mapped 1000 points per dance, which allowed us to calculate the proportion of points that fell inside of prairies and thus the probability that each dance advertised a flower patch within the reconstructed prairies.

#### 2.6.4 Analyzing the number of waggle runs per dance

Waggle dances often include multiple bouts, each involving some number of consecutive circuits (waggle runs and return phases), separated by periods of exchanging food or walking to a different area of the dance floor. Therefore, to determine foragers’ assessments of the profitability of prairie and non-prairie nectar sources, we tracked nectar dancers recorded at Belwin Conservancy back to the first frame in which the dancer entered the hive and then forward to the last frame in which the dancer was visible. In 352 of 401 cases, we could track the nectar dancer from the time that she entered the dance floor area until the time that she left it. We counted the total number of waggle runs in all bouts of those dances.

### 2.7 Pollen load collection and identification

To assess whether honey bees advertised native prairie pollens and determine which taxa they advertised, we took pollen loads from a subset of dancing foragers. During one of the days of video recording each week, we replaced the observation hive glass with a piece of plexiglass that had four 8–10 cm-diameter circular holes drilled in it. Each hole had a piece of transparent acetate sheet taped over it so that we could lift the sheet like a door. To collect data on the largest number of different flower patches, when we observed a dancer with pollen loads we categorized her dance by the angle (to the nearest 30 degree mark) and color of the pollen load (different pollen colors usually indicate different flower species, [65]). Honey bee foragers rarely collect pollen from more than one species of flowering plants on a given foraging trip [66]. Any time a dance for a new angle/color combination occurred, we treated it as representing a unique patch of flowers. For each unique patch, we allowed one dancer to complete 6 circuits and then captured her using a tubular mesh cage, which we placed around her until she walked into it. We then capped the cage and placed it on ice. As soon as the dancer was incapacitated by the cold temperatures (5–10 min), we briefly removed her from the cage and collected one of her pollen loads for later analysis. After the 50-minute recording period was complete, we placed caged foragers outside and allowed them to fly back to their colonies.

To make pollen identification easier by removing pollen contents, lipids, and other debris, we acetolyzed all pollen loads, a process that involves exposure to a heated mixture of acids [49]. We then examined the remaining sporopollenin walls of the pollen grains with a light microscope at 400x magnification and compared them to reference pollen slides from known species. Each time that we encountered new species/genera during floral surveys at Belwin Conservancy, we collected pollen and added it to our pollen reference library. When possible, we assigned the pollen taxa to the following categories: pollen from taxa native to prairie ecosystems and known to be present in the reconstructed prairies (S1 Table, S1 Fig, [67]), pollen from taxa native to the Upper Midwest region but not prairie ecosystems (ex. trees, vines, woodland forbs), and pollen from taxa that are not native to the Upper Midwest (based on the USDA PLANTS database plants.sc.egov.usda.gov). We decoded the dances and mapped the sources of these pollen types.

Of the 335 collected pollen loads, we cut 104 in half so that we could examine one half under the microscope and sequence the ITS (ribosomal internal transcribed spacer) region of the other half (primers: ITS4a and ITS5, [68]) to confirm microscopy IDs and provide more specific taxonomic identification in some cases. In cases where the ITS BLAST results indicated fungal taxa, we also sequenced a chloroplast gene, *rbcL* (RuBisCO large subunit; primers were according to [69]). We extracted DNA using 5 mm borosilicate beads, a Qiagen TissueLyser II, and Qiagen DNeasy Plant Mini-Kits. We amplified DNA markers using GE Healthcare Life Sciences illustra PuReTaq Ready-To-Go PCR Beads with 1 μl of DNA, 2.5 ul of 10nM of each primer, and 19 μl of water added, with thermocycler settings according to [68] for the ITS region and [69] for rbcL. Sanger sequencing was performed on an Applied Biosystems 3730xl DNA Analyzer. We cleaned the resulting sequences in Geneious and BLASTed them against the NCBI database. We assigned pollen loads to taxa based on the lowest taxonomic level shared by the top 10 BLAST hits for ITS (or in 10 cases, *rbcL*). We deposited sequencing results produced in this study into the GenBank database under accession numbers MN273343-MN273438 (ITS sequences) and MN284913-MN284922 (*rbcL* sequences).

### 2.8 Statistical analyses

#### 2.8.1 Proportion of dances advertising sites within prairies across the foraging season

We performed all statistical tests using R statistical software (version 3.6.0). After mapping 1000 points per dance, we calculated an Agresti-Coull 95% confidence interval [70,71] for the proportion of dances within prairies, where p is the number of dances within prairies and n is the total number of dances (in both cases scaled back to the actual number of dances by dividing the number of simulated points by 1000). In addition, we used Pearson’s chi-squared tests (chisq.test) to test whether the proportions of dances for prairie sites differed among parts of the foraging season (May/June, July, and August/September) at each site and for each food type (pollen and nectar) with a Bonferroni correction for multiple tests. When chi-squared tests showed a significant effect of season, we used mixed model logistic regressions with season as the fixed effect and colony as a random effect, weighted by the number of dances (glmer(Proportions_in_prairies ~ Season + (1|Colony), family = binomial(link = “logit”), weights = Dances), to get p-values for comparisons between parts of the season.

#### 2.8.2 Relationship between the probability that a nectar dance advertised sources within prairies, season, and the number of waggle runs per dance

For dances advertising nectar sources at Belwin Conservancy, we assessed the relationship between the number of waggle runs per dance, the part of the season, and the probability that a dance advertised a flower patch within a prairie using a linear model (lm). Because there was no significant effect of whether a dance advertised a prairie site on number of waggle runs that the dancer performed, we further analyzed the influence of season alone using an ANOVA (aov) and a Tukey honestly significant difference test (TukeyHSD).

#### 2.8.3 Seasonal changes in the average distance of advertised nectar sources

We calculated distances of advertised nectar sources using the average relationship between duration and distance from calibration data (see equation in section 2.5.3) and compared these distances using an ANOVA (aov). We evaluated differences in average distance advertised among parts of the foraging season using a Tukey HSD test (TukeyHSD).

## 3 Results

### 3.1 Dances advertising sites within prairies across the foraging season

**Fig 2:**
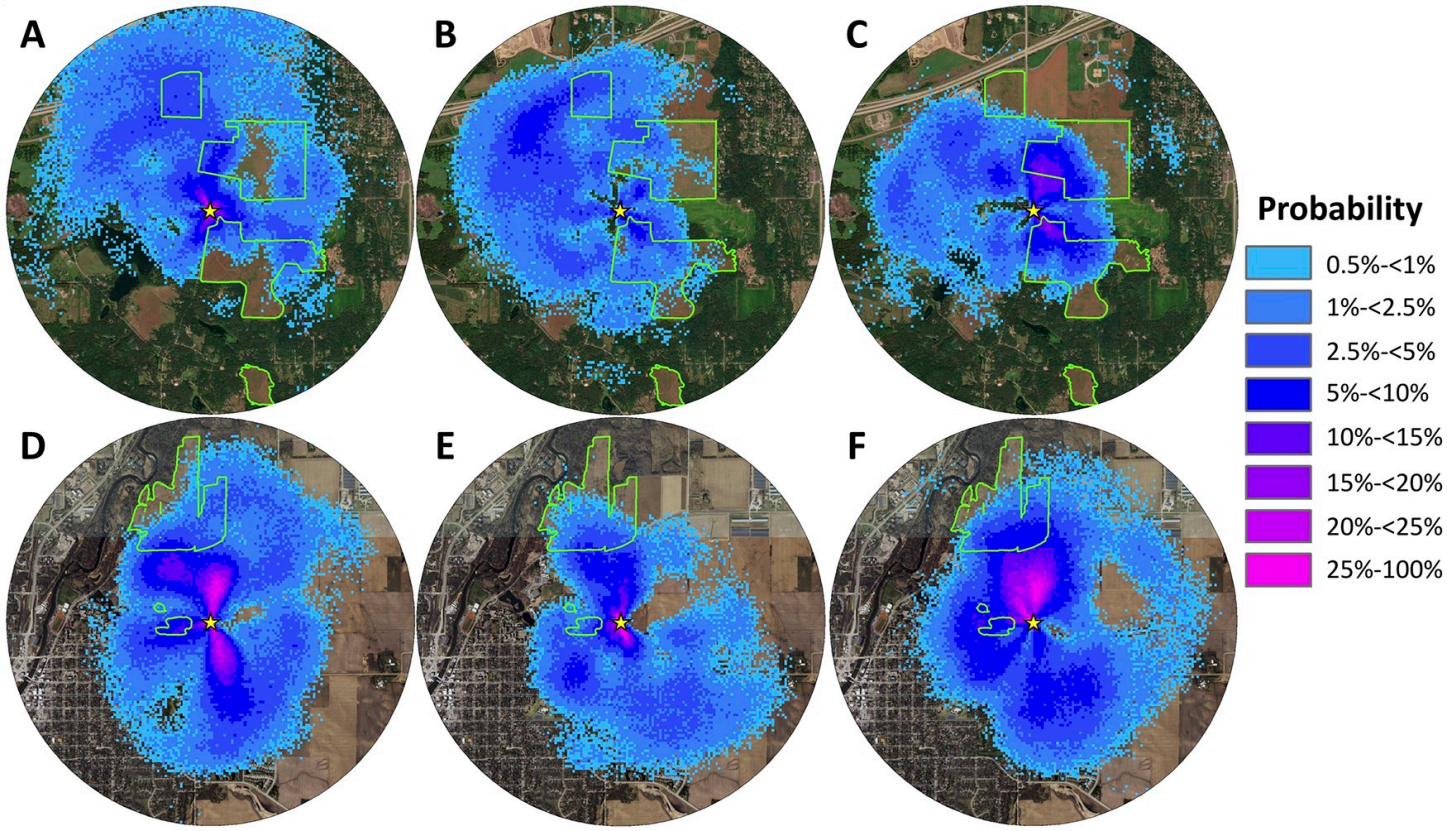
Probability density distributions of waggle dances at Belwin Conservancy (A: May/June, n = 246; B: July, n = 188; and C: August/September, n = 190) and at Carleton College (D: May/June, n = 329; E: July, n = 198; and F: August/September, n = 377). Colors show the probability that at least one dance advertised a flower patch within that 5 m x 5 m grid square. Green outlines show the edges of the restored prairies at each site. Yellow stars represent observation hive locations. Maps extend 2 km around observation hives.

**Fig 3:**
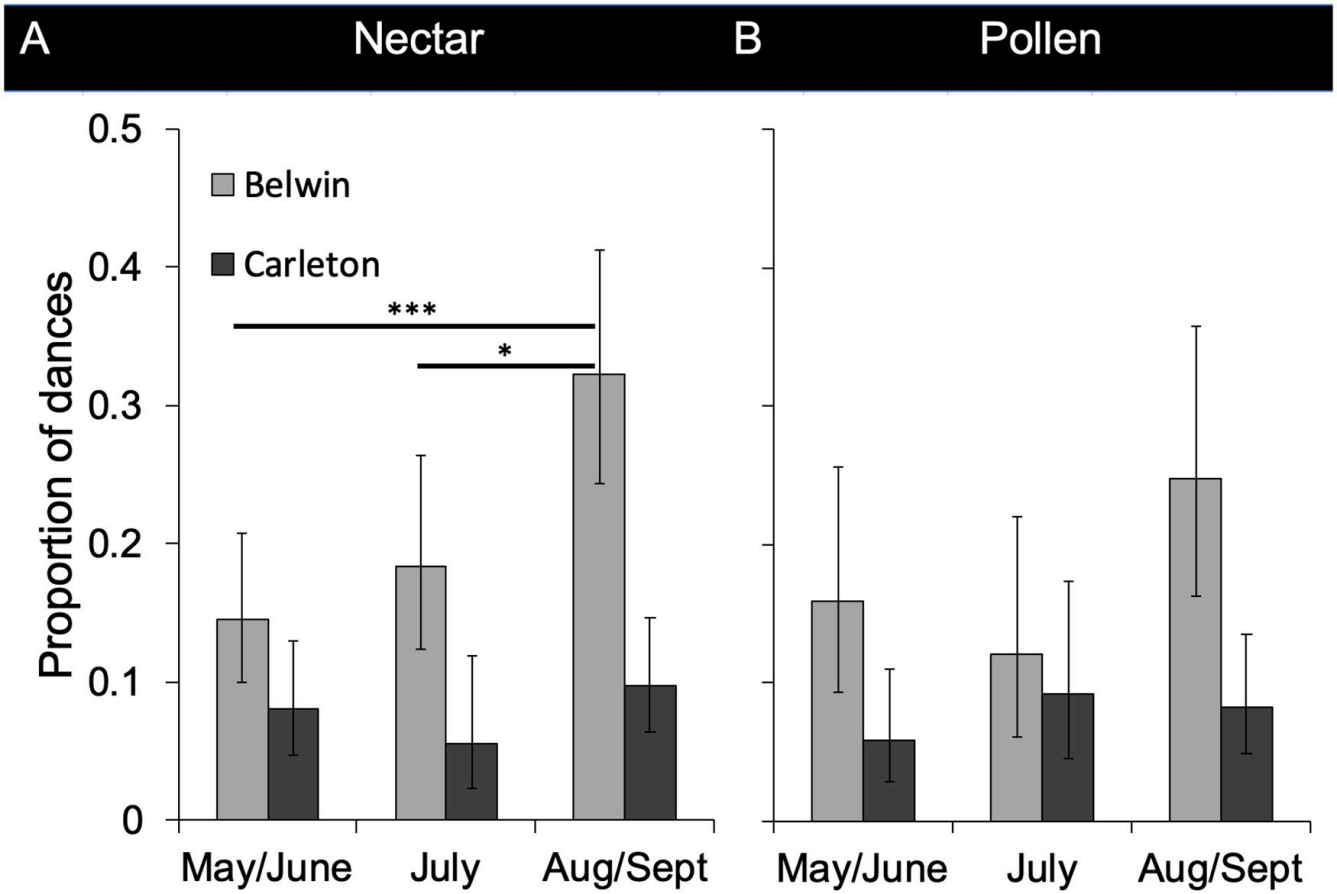
The estimated proportions of waggle dances advertising food sources within prairies at Belwin Conservancy (light gray) and Carleton College (dark gray). A) Nectar sources at Belwin Conservancy (May/June: n = 166 dances, July: n = 119, Aug/Sept: n = 116) and Carleton College (May/June: n = 180 dances, July: n = 109, Aug/Sept: n = 207). B) Pollen sources at Belwin Conservancy (May/June: n = 80 dances, July: n = 69, Aug/Sept: n = 74) and Carleton College (May/June: n = 149 dances, July: n = 89, Aug/Sept: n = 170). Proportions are based on locations of simulated points. Error bars show Agresti-Coull 95% confidence intervals, where p is the number of dances within prairies and an n is the total number of dances scaled back to the actual number of dances (number of simulated locations divided by 1000). * indicates p<0.05, *** indicates p<0.001. World Imagery base map layer sources: Esri, DigitalGlobe, GeoEye, i-cubed, USDA FSA, USGS, AEX, Getmapping, Aerogrid, IGN, IGP, swisstopo, and the GIS User Community.

At Belwin Conservancy, the estimated proportions of dances that advertised nectar sources within prairies were below 20% until August/September. In August and September, an estimated 32.2% of dances advertising nectar sources were for sites within reconstructed prairies (Figs 3 and 2), showing a statistically significant seasonal change (Fig 3; Table 1). A mixed model logistic regression analysis showed significant differences between May/June and August/September (z = -3.57, p<0.001) and between July and August/September (z = -2.48, p = 0.013), but not between May/June and July (z = -0.91, p = 0.36). There was a similar seasonal trend for the proportion of dances advertising pollen sources at Belwin Conservancy with an estimated 15.9% of dances advertising sites in prairies in May/June, 12.0% in July, and 24.8% in August/September, although it was not significant (Fig 3; Table 1). In contrast, the proportion of dances advertising both nectar and pollen sources in prairies at Carleton College was estimated at less than 10% throughout the foraging season and did not change significantly from month to month (Fig 3; Table 1). The error in the distance and directional information within the dances may have affected our estimates of the proportion of dances that advertised prairie sites somewhat differently at the two sites. It is possible that our estimates at Carleton College were lower than at Belwin Conservancy in part due the smaller size of the closest prairie parcels at Carleton College. Despite this potential bias, our sample size of 904 dances should still have been sufficient to reveal a seasonal trend at Carleton College.

**Table 1:** Pearson’s Chi-squared tests to determine whether the proportion of dances that advertised food sources within prairies changed over the course of the season (df = 2).

Site	Food type	Chi-squared value	P-value
Belwin Conservancy	Nectar	13.15	0.0014 **
Belwin Conservancy	Pollen	4.15	0.13
Carleton College	Nectar	01.08	0.58
Carleton College	Pollen	02.44	0.30

**Fig 4:**
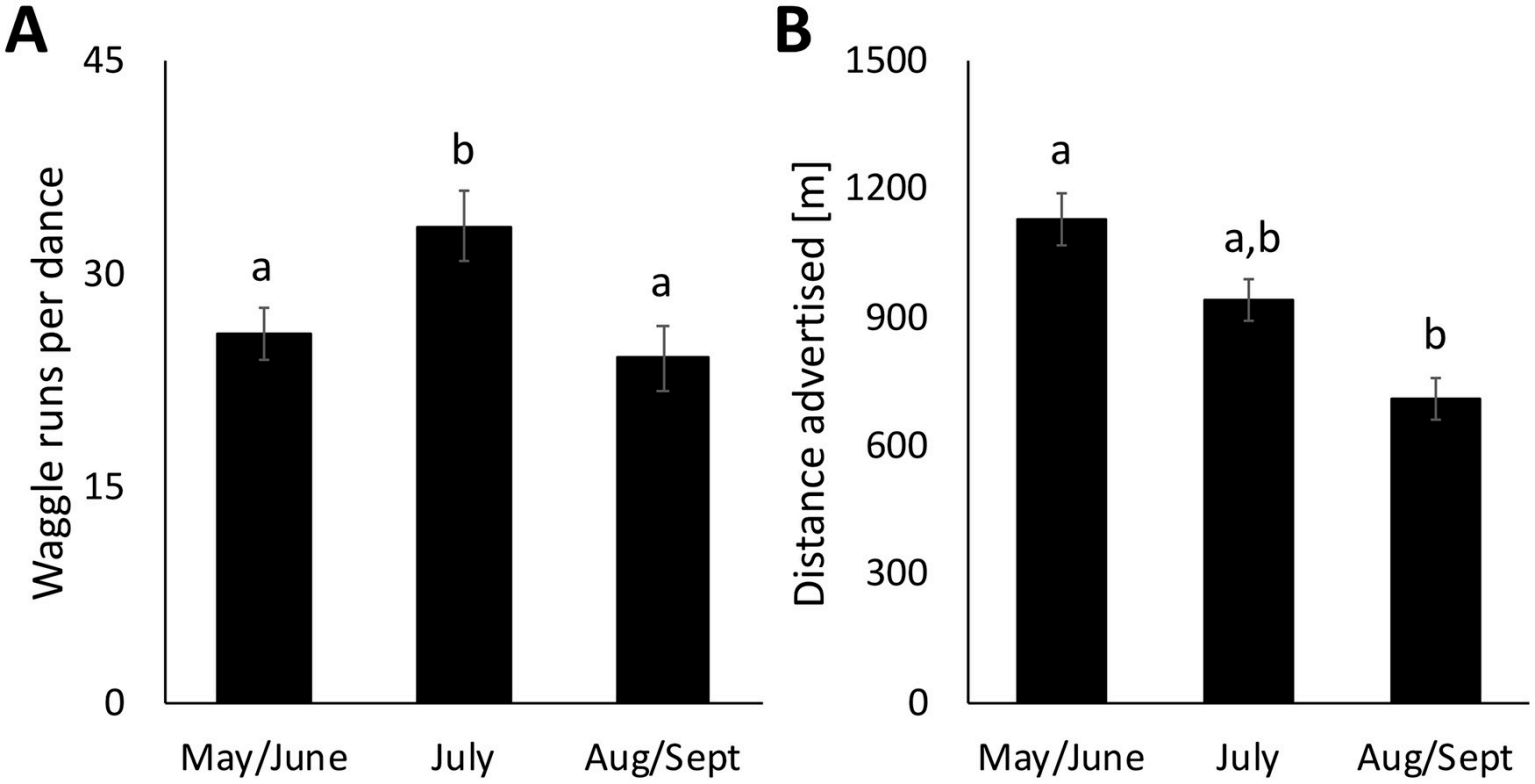
A comparison across the foraging season of the mean number of (A) waggle runs per dance and (B) the mean distances [m] advertised in dances for nectar sources at Belwin Conservancy. Error bars show the standard error of the mean. Letters indicate significant differences (p<0.05). Sample sizes: waggle runs- May/June (n = 147), July (n = 106), and August/September (n = 99); distances- May/June (n = 166), July (n = 119), and August/September (n = 116).

To better understand the seasonal change at Belwin Conservancy, we looked at whether there were differences between dances that were likely advertising prairie sites and dances that were likely not advertising prairie sites (proportion of simulated points that fell within prairies). Specifically, we looked at the number of times that the dancer repeated the location information during her dance (total number of waggle runs performed). We found no significant effect of the probability that a given nectar dance advertised a prairie site on the total number of waggle runs in that dance (df = 346, t = 0.734, p = 0.46) nor any interaction between season and probability that a dance advertised a prairie site (p = 0.30 and p = 0.46). However, we did see a significant effect of season on dance length both in the model with interactions (t = 2.86, p<0.01) and in a subsequent ANOVA with only season as a fixed effect (F_(2,349)_ = 4.84, p<0.01; Fig 4). Dances in July (33.43±3.22 waggle runs, mean±SEM) included a significantly greater number of waggle runs than dances in May/June (25.92±3.00 waggle runs; p<0.05) or August/September (24.23±2.32 waggle runs; p<0.05).

We also compared the average distances of advertised nectar sources across the foraging season. At Belwin Conservancy, dancers advertised nectar sources that were an average of 954.27±33.73 meters (mean±SEM) away from the hive, with a maximum advertised distance of 4,873.61 meters. However, there was a significant decrease across the season from an average of 1,129.72±62.24 in May and June to an average of 713.30±49.62 in August and September (F_(2,398)_ = 13.83, p<0.0001; Fig 4). At Carleton College, the average distance of advertised nectar sources was 817.57±34.73 and did not change significantly across the season (F_(2,381)_ = 0.45, p = 0.64).

The averages and trends for dances advertising pollen sources were very similar to those for nectar sources. Pollen dances at Belwin Conservancy advertised sources at an average distance of 927.35±40.84 meters with a significant seasonal decrease from May to August (F_(2,220)_ = 5.79, p<0.01). Pollen dances at Carleton College advertised an average distance of 830.91±32.56 meters with no significant seasonal change (F_(2,329)_ = 0.80, p = 0.45).

### 3.2 Taxa of advertised pollen sources

**Fig 5:**
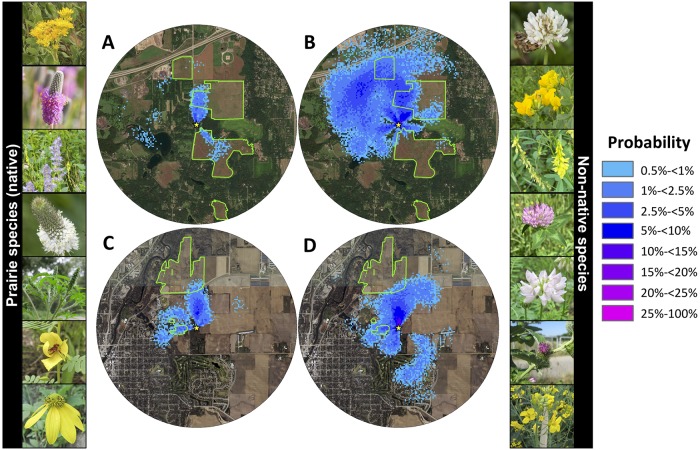
Probability density distributions of waggle dances advertising prairie pollen sources at Belwin Conservancy (A; n = 15 dances) and Carleton College (C; n = 19 dances) and non-native pollen sources at Belwin Conservancy (B; n = 129 dances) and Carleton College (D; n = 59 dances). Colors show the probability that at least one dance advertised a flower patch within that 5 m x 5 m grid square. Green outlines show the edges of the restored prairies at each site. Yellow stars represent observation hive locations. Maps extend 2 km around observation hives. Photos on left show all confirmed native prairie taxa advertised: *Solidago* sp.*, Dalea purpurea*, *Agastache* sp.*, Dalea candida*, *Ambrosia* sp., Chamaecrista fasciculata*, and a member of the tribe Heliantheae (probably *Rudbeckia* sp.*). Photos on right show the top seven taxa of non-native flowers advertised: Trifolium repens/hybridum*, Lotus corniculatus*, Melilotus officinalis*, Trifolium pretense/incarnatum*, Securigera varia*, *Arctium minus*, and *Brassica* sp. *Image credits: Heather Holm. World Imagery base map layer sources: Esri, DigitalGlobe, GeoEye, i-cubed, USDA FSA, USGS, AEX, Getmapping, Aerogrid, IGN, IGP, swisstopo, and the GIS User Community.

Foragers in this study were recorded dancing to recruit nestmates to collect pollen from at least 34 different genera in 25 families, including seven native prairie taxa (Fig 5; S1 Table). Dancers advertised these native prairie taxa in July, August, and September (S1 Table). Of the 335 pollen loads collected, 9.2% were from native prairie taxa, 9.9% were from taxa native to the region but from non-prairie ecosystems (forests, wetlands, etc.), 58.8% were from non-native taxa, and 22.1% could not be identified specifically enough to determine whether they were native to the region or not (Fig 5; S1 Table). Flower surveys documented 21 species of native prairie flowers blooming at Belwin Conservancy and 10 at Carleton College (S1 Fig). These included five of the seven native prairie taxa advertised, including *Solidago* spp., *Agastache* spp., *Dalea candida*, *Dalea purpurea*, and *Rudbeckia* spp.

## 4 Discussion

The results of our study provided answers to two main questions about how honey bee foragers behave when given access to large reconstructed prairies. First, although most dances advertised non-prairie food sources, at Belwin Conservancy, we saw a significant increase in the proportion of dances for nectar sources within prairies at the end of the foraging season. Second, we determined that honey bee foragers do perceive seven taxa of native prairie flowers as profitable pollen sources and advertise them with dances: *Solidago* spp., *Dalea purpurea*, *Agastache* sp., *Dalea candida*, *Ambrosia* spp., *Chamaecrista fasciculata*, and a member of the tribe Heliantheae (probably a *Rudbeckia* species based on the top BLAST hits).

To better understand the seasonal changes in the proportion of nectar dances advertising prairie sites at Belwin Conservancy, we determined whether foragers advertising nectar sources in prairies performed more or fewer waggle runs than foragers advertising nectar sources outside of prairies across the foraging season. We found no significant relationship between the probability that a dance advertised a prairie site and the number of waggle runs performed. A significantly greater number of waggle runs for nectar sources within prairies in August/September would have supported the idea that foragers perceived late season prairie flowers as more profitable sources of nectar than late season non-prairie flowers. The lack of difference suggests that the increased proportion of dances in August/September may instead reflect changes in the abundance of rewarding flowers inside of prairies relative to other areas. However, we did see a significant effect of season, with July having the longest dances. The greater number of waggle runs indicates that foragers invested more energy into recruiting other foragers during this period, which could result from foragers perceiving July nectar sources as more profitable or perceiving a greater colony need for or ability to store nectar during July [72]. Recruits that follow more waggle runs are significantly more likely to find a food source [73] so an average increase of 7–9 waggle runs in July could potentially lead to an average higher recruitment success per dance. Because each recruit will likely also dance, this per-dance difference in waggle runs would likely increase recruitment to that site exponentially as more foragers advertise it [74].

We also looked at the average distances of advertised nectar sources across the season at Belwin Conservancy and found a significant seasonal change with shorter distances advertised in the later season. The average distances that we found are within the range of previous studies [36,43,71,75]. However, the decrease in distances advertised is surprising because multiple studies have indicated that honey bee colonies in the Upper Midwest and surrounding states face a dearth period when there is a lower diversity of blooming flowers at the end of the summer and into the fall (North Dakota: [76]; Ohio: [75]; Michigan: [77]; Iowa: [78]). The period from the beginning of July to early August is generally the time of year when honey bee colonies in Minnesota gain the most weight and produce the most honey [79,80]. A nectar dearth period generally results in longer distances advertised [27,40,41,42]. Our results suggest that being close to a reconstructed prairie in the later season may provide a significant fitness benefit to colonies by allowing them to forage closer to home and expend less energy per trip at a time of year when they might otherwise have needed to travel much farther. A recent study found that colonies in landscapes dominated by corn and soybeans lose weight and their nurse bees lose fat stores starting in August but moving colonies to a large prairie in August rescued them from these effects [78]. The lower flight distances that we saw in August/September could potentially contribute to this rescue effect.

There are several possible explanations for why foragers in our colonies showed a seasonal change in the proportion of waggle dances for nectar sources in prairie sites at Belwin Conservancy but not Carleton College. Differences in the weather between the two sites may have affected nectar production of native prairie and non-prairie species differently. Both temperature and rainfall can have significant effects on the flowering time and nectar production of many species of forbs [81,82,83]. The average distance between our colonies and the reconstructed prairies at Belwin Conservancy was smaller than the average distance between our colonies and the reconstructed prairies at Carleton College (Fig 1), which would make them easier to fly to and, thus, likely more attractive [71]. We also recorded a greater diversity of flowering plants at Belwin Conservancy, including *Solidago rigida*, blooming in the later season, which may have been particularly attractive sources of both nectar and pollen at that time of year (S1 Fig). Unfortunately, our pollen identification methods cannot differentiate between species in the genus *Solidago*, but they did reveal the genus as one of the most commonly advertised native prairie pollen sources (S1 Table). *Solidago* species tend to grow in large, dense clusters due to their ability to produce colonies of clones using rhizomes [84]. This growth pattern may make patches of *Solidago* particularly attractive resources for honey bees given that many foragers can be recruited to the same patch of flowers without depleting it of food. It is possible that the *Solidago* plants around Belwin Conservancy were more concentrated within the reconstructed prairies while a larger proportion of the *Solidago* plants blooming near our colonies at Carleton College were growing on roadsides or in other non-prairie habitats. Given that we were only able to survey flowers within prairies, we cannot be certain.

In addition to the seven prairie taxa, foragers in our study advertised a diverse set of non-native and native pollens from non-prairie habitats. The three most commonly advertised pollen taxa were *Trifolium repens/hybridum*, *Melilotus officinalis*, and *Lotus corniculatus* (S1 Table). These taxa are non-native forbs from Europe brought to North America as forage crops for livestock, and they have since become established as common weeds [85]. All three have a long bloom period centered around July (S1 Fig), which may help to explain the large number of patches advertised. The fact that honey bees were also brought to North America from Europe raises the question of whether honey bees may have evolved strong preferences for cues from flowers in their native range. On the other hand, the success of European honey bees on six continents and the fact that honey bees are the most frequently recorded floral visitors in natural habitats across the globe [86] suggests that they have very plastic foraging preferences. Dances advertised a number of other non-native weedy taxa, including *Trifolium pratense/incarnatum*, *Securigera varia*, *Arctium minus*, and *Brassica* spp. (S1 Table). These non-native taxa match taxa found in pollen collected by honey bee colonies in a separate nearby study, except for *Arctium minus* and *Lotus corniculatus* [13,76]. Maps of dances advertising them indicate that most, but not all, dances for those taxa were for sites outside of prairies (Fig 5). Flower surveys confirmed that a number of non-native species bloomed in prairies at both sites (S1 Fig). In addition, dances in May, June, and July advertised a number of native non-prairie taxa, including woody species in genera such as *Gleditsia*, *Rhus*, *Cornus*, *Tilia*, and *Parthenocissus* that provide honey bee foragers with very concentrated patches of flowers (S1 Table).

## 5 Conclusion

While we did not find evidence that reconstructed prairies provide a highly attractive resource for honey bees in May, June, and July, we did find evidence that reconstructed prairies can become very attractive in the later season, potentially leading to significant health benefits to honey bee colonies. In addition, we found that honey bee foragers perceived seven native prairie taxa found in reconstructed prairies as worth advertising to their nestmates. Our results suggest that including these taxa, especially *Dalea purpurea*, *Dalea candida*, and *Agastache* sp. at high densities may make prairies more attractive to honey bees in July, which is useful information for both land managers who want to provide food for honey bees and those that want to avoid potential competition between honey bees and native bees on their land. In cases where landowners are concerned about competition [87], they may either want to avoid planting these species at high densities, or, if their goal is to conserve specialist bees that rely on those species for pollen (ex. *Colletes* specialists on *Dalea* [88]), they may want to limit the number of honey bee colonies with access to the prairie planting. In addition, our results highlight the current importance of several species of non-native pollen sources, including species in the genus *Trifolium*, *Melilotus officinalis*, and *Lotus corniculatus* to honey bee colonies. We are currently examining the full diets of honey bee colonies located near reconstructed prairies to provide more information about the most attractive prairie species for honey-bee friendly plantings. Future experiments involving planting carefully-controlled patches of flowers and looking at honey bee recruitment behavior could help to determine how important both patch area and planting density are in attracting honey bee foragers.

## Supporting information

S1 Data Durations of dances for syrup feeders at different distances from the observation hives at Belwin Conservancy in 2015. (CSV)

S2 Data A comma separated values file of data from 1528 waggle dances decoded at Belwin Conservancy and Carleton College. (CSV)

S3 Data A comma separated values file of the total number of waggle runs in dances for nectar sources at Belwin Conservancy. (CSV)

S4 Data A comma separated values file of the estimated distances of nectar and pollen sources advertised by foragers at Belwin Conservancy. (CSV)

S5 Data A comma separated values file of the pollen identifications and dance decoding data from both sites. (CSV)

S6 Data A comma separated values file of sites, dates, and the proportion of surveyed quadrats in which we observed each forb species. (CSV)

S1 Table Pollen loads collected from waggle-dancing foragers. Pollen loads were collected from each dance advertising a unique pollen color/dance angle combination to determine the taxon of each flower patch advertised during the 50-minute weekly sampling period. Native prairie taxa are marked with an asterisk symbol (*) and non-native taxa are marked with a plus symbol (+).
(DOCX)

S1 Fig Range of dates when forb species were observed blooming at Belwin Conservancy (A) and Carleton College (B). Native prairie species are marked with an asterisk symbol (*) and non-native species are marked with a plus symbol (+). Surveys were conducted once every two weeks throughout the period that honey bee colonies were video recorded. All forb species blooming within fifty 1-meter quadrats placed randomly on five 200-meter transects within the restored prairies at each site were identified and recorded. Therefore, species may have had a longer blooming period than recorded in surveys.
(DOCX)
