# Correction: Do honey bee (*Apis mellifera*) foragers recruit their nestmates to native forbs in reconstructed prairie habitats?

**DOI:** 10.1371/journal.pone.0259603

**Published:** 2021-11-01

**Authors:** Morgan K. Carr-Markell, Cora M. Demler, Margaret J. Couvillon, Roger Schürch, Marla Spivak

In section 2.6.3 of the Materials and methods, there is an error in the estimated relationship between waggle run duration and distance advertised, taken from a linear regression of the combined calibration datasets collected during this study and a previous study (Schürch et al. 2013). The correct estimated relationship is: D = 809.4d – 206.5.

This discrepancy was due to an error in copying the coefficients, which then caused the converted slope to be 14.3 m higher and the intercept to be 20.1 m lower than they should have been. Due to the error, there are also errors in the third and fourth paragraphs of section 3.1 of the Results. The correct paragraphs are:

We also compared the average distances of advertised nectar sources across the foraging season. At Belwin Conservancy, dancers advertised nectar sources that were an average of 953.86±33.14 meters (mean±SEM) away from the hive, with a maximum advertised distance of 4,805.06 meters. However, there was a significant decrease across the season from an average of 1,126.26±61.16 in May and June to an average of 717.07±48.76 in August and September (F(2,398) = 13.83, p<0.0001; Figure 4). At Carleton College, the average distance of advertised nectar sources was 806.91±29.02 and did not change significantly across the season (F(2,493) = 0.37, p = 0.69).

The averages and trends for dances advertising pollen sources were very similar to those for nectar sources. Pollen dances at Belwin Conservancy advertised sources at an average distance of 927.40±40.13 meters with a significant seasonal decrease from May to August (F(2,220) = 5.79, p<0.01). Pollen dances at Carleton College advertised an average distance of 797.83±26.47 meters with no significant seasonal change (F(2,405) = 0.89, p = 0.41).
