# Supplementary material for: Do honey bee (Apis mellifera) foragers recruit their nestmates to native forbs in reconstructed prairie habitats?
Source: PLoS One. 2020 Feb 12;15(2):e0228169. doi: 10.1371/journal.pone.0228169 (PMC7015315; doi:10.1371/journal.pone.0228169)
Supplement: S1 Table — Pollen loads were collected from each dance advertising a unique pollen color/dance angle combination to determine the taxon of each flower patch advertised during the 50-minute weekly sampling period. Native prairie taxa are marked with an asterisk symbol (*) and non-native taxa are marked with a plus symbol (+). (DOCX) [file pone.0228169.s007.docx]

**Table S1**

Pollen loads collected from waggle-dancing foragers. Pollen loads were collected from each dance advertising a unique pollen color/dance angle combination to determine the taxon of each flower patch advertised during the 50-minute weekly sampling period. Native prairie taxa are marked with an asterisk symbol (*) and non-native taxa are marked with a plus symbol (+).

| Site | Month | Pollen taxa | Number of advertised patches |
| --- | --- | --- | --- |
| Belwin Conservancy | May/June | Species: *Trifolium repens/hybridum+*  Species: *Lotus corniculatus+*  Genus: *Rhamnus+*  Species: *Melilotus officinalis+*  Species: *Gleditsia triacanthos*  Family: Rosaceae  Family: Rutaceae  Family: Brassicaceae  Species: *Trifolium pretense/incarnatum+*  Species: *Securigera varia*+  Genus: *Acer*  Genus: *Elaeagnus*  Family: Poaceae (not *Zea mays*)  Species: *Acer tataricum+*  Species: *Digitalis lanata+*  Genus: *Brassica+*  Genus: *Celastrus*  Genus: *Cornus*  Genus: *Prunus*  Genus: *Quercus*  Tribe: Mentheae  Mixed load- Families Asteraceae/Rosaceae  Unknown | 16  13  8  5  4  4  4  3  3  2  2  2  2  1  1  1  1  1  1  1  1  1  2 |
|  | July | Species: *Lotus corniculatus+*  Species: *Trifolium repens/hybridum+*  Species: *Melilotus officinalis+*  Species: *Securigera varia+*  Species: *Trifolium pretense/incarnatum+*  Species: *Arctium minus+*  Genus: *Agastache**  Genus: *Brassica+*  Genus: *Tilia*  Genus: *Solidago**  Genus: *Carduus+*  Genus: *Centaurea+*  Family: Brassicaceae  Genus: *Parthenocissus*  Genus: *Lonicera*  Tribe: Mentheae  Family: Apiaceae (not *Zizia*)  Unknown | 17  8  5  5  5  4  4  4  3  2  2  2  2  1  1  1  1  3 |
| Belwin Conservancy | August/September | Species: *Melilotus officinalis+*  Family: Asteraceae  Genus: *Solidago**  Species: *Lotus corniculatus+*  Species: *Trifolium repens/hybridum+*  Genus: *Alisma*  Species: *Trifolium pretense/incarnatum+*  Genus: *Rhus*  Genus: *Agastache**  Genus: *Ambrosia**  Genus: *Brassica*+  Genus: *Taraxacum+*  Genus: *Tanacetum+*  Tribe: Anthemideae  Tribe: Cichorieae  Family: Rosaceae  Unknown | 10  10  7  7  7  6  4  3  1  1  1  1  1  1  1  1  16 |
| Carleton College | May/June | Species: *Trifolium repens/hybridum+*  Genus: *Rhus*  Genus: *Brassica+*  Species: *Lotus corniculatus+*  Species: *Securigera varia*+  Tribe: Anthemideae  Sub-family: Apoideae (not *Zizia*) | 7  3  2  1  1  1  1 |
|  | July | Species: *Trifolium repens/hybridum+*  Species: *Lotus corniculatus+*  Species: *Dalea purpurea**  Species: *Dalea candida**  Species: *Arctium minus+*  Species: *Securigera varia+*  Genus: *Brassica+*  Genus: *Coriandrum+*  Genus: *Parthenocissus*  Genus: *Potentilla*  Genus: *Rhus*  Genus: *Silene*  Sub-family: Apoideae (not *Zizia*)  Mixed load- Genera *Tilia*/*Trifolium* | 13  8  5  4  4  2  2  2  2  1  1  1  1  1 |

| Carleton College | August/September | Species: *Trifolium repens/hybridum+*  Genus: *Impatiens*  Genus: *Solidago**  Species: *Lotus corniculatus+*  Genus: *Hydrangea*  Genus: *Carduus+*  Genus: *Taraxacum*+  Family: Asteraceae  Species: *Dalea purpurea**  Species: *Chamaecrista fasciculata**  Genus: *Agastache**  Genus: *Ambrosia**  Tribe: Heliantheae*  Species: *Securigera varia+*  Species: *Melilotus officinalis+*  Species: *Arctium minus*+  Family: Araliaceae  Family: Scrophulariaceae  Unknown | 6  6  5  4  3  2  2  2  1  1  1  1  1  1  1  1  1  1  5 |
| --- | --- | --- | --- |
