# Supplementary material for: Do honey bee (Apis mellifera) foragers recruit their nestmates to native forbs in reconstructed prairie habitats?
Source: PLoS One. 2020 Feb 12;15(2):e0228169. doi: 10.1371/journal.pone.0228169 (PMC7015315; doi:10.1371/journal.pone.0228169)
Supplement: S1 Fig — Native prairie species are marked with an asterisk symbol (*) and non-native species are marked with a plus symbol (+). Surveys were conducted once every two weeks throughout the period that honey bee colonies were video recorded. All forb species blooming within fifty 1-meter quadrats placed randomly on five 200-meter transects within the restored prairies at each site were identified and recorded. Therefore, species may have had a longer blooming period than recorded in surveys. (DOCX) [file pone.0228169.s008.docx]

**
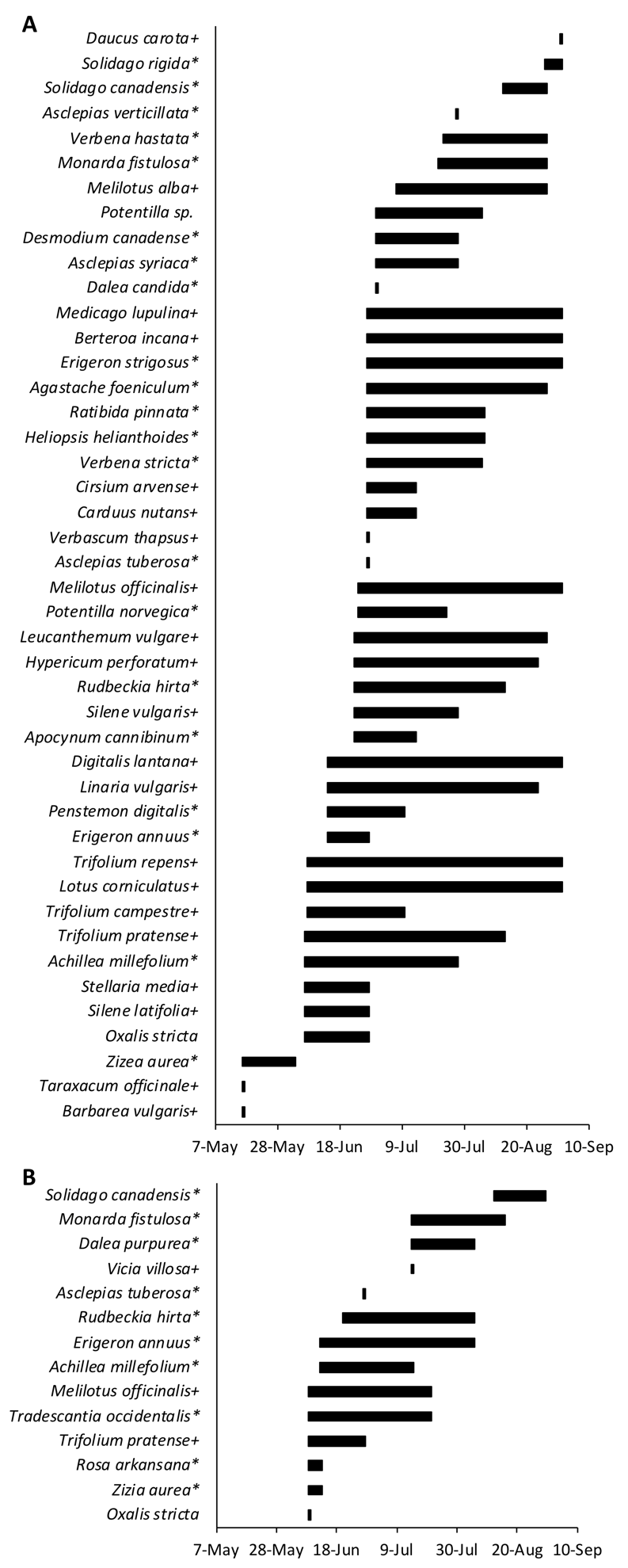
 Fig. S1**. Range of dates when forb species were observed blooming at Belwin Conservancy (A) and Carleton College (B). Native prairie species are marked with an asterisk symbol (*) and non-native species are marked with a plus symbol (+). Surveys were conducted once every two weeks throughout the period that honey bee colonies were video recorded. All forb species blooming within fifty 1-meter quadrats placed randomly on five 200-meter transects within the restored prairies at each site were identified and recorded. Therefore, species may have had a longer blooming period than recorded in surveys.
